# Supplementary material for: VO2max prediction based on submaximal cardiorespiratory relationships and body composition in male runners and cyclists: a population study
Source: eLife. 2023 May 10;12:e86291. doi: 10.7554/eLife.86291 (PMC10198721; doi:10.7554/eLife.86291)
Supplement: Source code 1. [file elife-86291-code1.zip › 18-01-2023-RA-eLife-86291/Python_Code.pdf]

```
import pandas as pd
import numpy as np
import xlrd
import os
import logging
import datetime

logging.basicConfig(filename='log.log', filemode='w', level=logging.INFO,
                    format='%(asctime)s %(message)s')

logging.info(f'INFO: sciezka {os.getcwd()}')
print(os.getcwd())

try:
    dirs = os.listdir('klienci\\')
except FileNotFoundError:
    logging.error('Błędna scieżka')
    logging.exception("message")
    print('Błędna sciezka')
    raise

def mleczan_udzial(df, prog, power):

    df1 = df[df['PRĘDKOŚĆ/MOC'] == power]
    prog_l = len(df1)

    if type(prog) == str:
        prog = pd.to_datetime(pd.Series(prog), format='%H:%M:%S').dt.time[0]

    df2 = df1[pd.to_datetime(df1.t,format= '%H:%M:%S' ).dt.time <= prog]
```

```
    pozycja = len(df2)

    poz_proc = float(pozycja)/float(prog_l)

    return poz_proc

def mleczan(df1, df3, prog, power):

    df3['Lag'] = df3['LA (mleczan)'].shift()
    df3['Diff'] = df3['LA (mleczan)'].diff()

    s3 = df3[df3['PRĘDKOŚĆ/MOC'] == power]
    try:
        s3['Udzial'] = mleczan_udzial(df1, prog, power)
    except Exception:
        logging.error('Błąd liczenia mleczanu')
        logging.exception("message")
        pass

    ml = s3.Lag + s3.Diff * s3.Udzial

    return ml.reset_index()[0]

def progi(df_1, df_3, prog_t, prog_opis):

    if prog_opis=='Max':
        s_1 = pd.DataFrame(df_1.max()).T
    else:
        s_1 = df_1[df_1.t == prog_t].iloc[0:1]
    s_1_dict = {
            'Speed':f'{prog_opis} - Prędkość biegu',
```

```
        'Power':f'{prog_opis} - Moc generowana (globalnie)',
        'VO2/Kg':f'{prog_opis} - Pobór tlenu- VO2 (relatywne)',
        'VO2':f'{prog_opis} - Pobór tlenu- VO2 (absolutne)',
        'HR':f'{prog_opis} - Tętno',
        'VE':f'{prog_opis} - Wentylacja płuc (VE)',
        'Rf':f'{prog_opis} - Częstość oddechów',
        'EEkg':f'{prog_opis} - Koszt fizjologiczny biegu',
        'koszt':f'{prog_opis} - Koszt fizjologiczny biegu',
        'koszt (ekonomia)':f'{prog_opis} - Koszt fizjologiczny biegu',
        'LA (mleczan)':f'{prog_opis} - Stężenie mleczanów (koniec obciążenia)'
        }

if 'Load1' in df_1.columns:
    load1_mean = df_1.Load1.mean()

    if load1_mean <= 30:  # = biegacz
        s_1_dict['Load1'] = f'{prog_opis} - Prędkość biegu'
    else:  # = kolarz
        s_1_dict['Load1'] = f'{prog_opis} - Moc generowana (globalnie)'

try:
    s_1['PRĘDKOŚĆ/MOC'] = s_1['PRĘDKOŚĆ/MOC'].astype(int)
except Exception:
    pass

if df_3 is not None:
    s_1 = s_1.merge(df_3, how='left', on='PRĘDKOŚĆ/MOC', suffixes=['x',''])

power = s_1['PRĘDKOŚĆ/MOC'].iloc[0]

list1 = ['Speed','Power','Load1','VO2/Kg','VO2','HR','VE','Rf','EEkg',
```

```
        'koszt','koszt (ekonomia)','LA (mleczan)']
    list2 = [a for a in list1 if a in s_1.columns.tolist()]
    s_1 = s_1[list2]
    s_1 = s_1.rename(columns=s_1_dict)

    if df_3 is not None:
        s_1[f'{prog_opis} - Stężenie mleczanów (na progu)'] = mleczan(df_1, df_3, prog_t, power)

    return s_1

def file1(klient):

    try:
        file = 'klienci\\'+klient+'\\1.xlsx'
        try:
            wb = xlrd.open_workbook(file)
            logging.info('INFO: Udało się otworzyć plik 1.xlsx')
        except Exception:
            logging.info('INFO: Nie mogę otworzyć pliku 1.xlsx')
            logging.exception("message")

        typ = 'xlsx'

        try:
            df_a = pd.read_excel('klienci\\'+klient+'\\1.xlsx', sheet_name='Wyniki', header=1)
        except Exception:
            logging.info('INFO: Nie mogę otworzyć arkusza Wyniki z pliku 1.xlsx')

        try:
            a_columns = df_a.iloc[df_a[df_a.Parametr.str.contains('Protok').fillna(False)].index[0]+1].tolist()
            a_dane = df_a.iloc[df_a[df_a.Parametr.str.contains('Protok').fillna(False)].index[0]+2].tolist()
```

```
        df_a = pd.DataFrame([a_dane], columns=a_columns)
    except Exception:
        logging.info('INFO: Nie mogę przetworzyć arkusza Wyniki z pliku 1.xlsx')
        logging.exception("message")

    AT = df_a.AT[0]
    RC = df_a.RC[0]
    Prog_Max = df_a['Max'][0]

except Exception:
    try:
        file = 'klienci\\'+klient+'\\1.xls'

        try:
            try:
                wb = xlrd.open_workbook(file)
                code = 0
            except Exception:
                wb = xlrd.open_workbook(file, encoding_override="cp1252")
                code = 1

            logging.info('INFO: Udało się otworzyć plik 1.xls')
        except Exception:
            try:
                file = file+'x'
                wb = xlrd.open_workbook(file)
                code = 0
                logging.info('INFO: Udało się otworzyć plik 1.xlsx w starym formacie')
            except Exception:
                logging.info('INFO: Nie mogę otworzyć pliku 1.xls')
                logging.exception("message")
```

```
            code = None

        typ = 'xls'

        sheet = wb.sheet_by_index(0)
        AT = sheet.cell_value(11, 7)
        RC = sheet.cell_value(12, 7)

        df_a = pd.read_excel(wb, engine='xlrd').iloc[2:,9:]
#          df_a = pd.read_excel(file).iloc[2:,9:]
        Prog_Max = df_a.t.max()
    except Exception:
        logging.error('Błąd: nie można znaleźć pliku 1')
        logging.exception("message")
        return

    sheet = wb.sheet_by_index(0)
    var = {}
    var['nazwisko'] = sheet.cell_value(1, 1)+' '+sheet.cell_value(2, 1)
    var['plec'] = sheet.cell_value(3, 1)
    var['AT'] = AT
    var['RC'] = RC
    var['Max'] = Prog_Max



    if typ == 'xlsx':
        var['data_testu'] = sheet.cell_value(0, 4)
        var['protokol'] = sheet.cell_value(7, 4)
    else:
        var['data_testu'] = sheet.cell_value(1, 4)
        var['protokol'] = np.nan
```

```
#    df = pd.read_excel(file).iloc[2:,9:]
    df = pd.read_excel(wb, engine='xlrd').iloc[2:,9:]

    var['df'] = df.copy()

    if 'Speed' in df.columns:
        var['Rodzaj testu'] = 'Biegacz'
    elif 'Power' in df.columns:
        var['Rodzaj testu'] = 'Kolarz'
    elif 'Load1' in df.columns:
        if df.Load1.mean()<=30:
            var['Rodzaj testu'] = 'Biegacz'
        else:
            var['Rodzaj testu'] = 'Kolarz'


    return var

def scanfile2(file):

    try:
        wb = xlrd.open_workbook(file)
    except Exception:
        logging.info('INFO: Nie mogę otworzyć pliku 2')
        logging.exception("message")

    sheet = wb.sheet_by_index(0)

    i = 0
    s = sheet.cell_value(i, 0)
```

```
    names = ['CUSTOMER', 'RISK', 'BMI', 'BODYFAT', 'TABC', 'BODYWATER']
    row = ''
    startrow = 0
    mapa = {}

    while ((s in names) or (type(s)==float) or (type(s)==int)):

        if s in names:
            row = s
            startrow = i
        else:
            mapa[row] = [int(s), startrow]

        i += 1
        try:
            s = sheet.cell_value(i, 0)
        except Exception:
            s = None

    return mapa

def file2(klient):

    file = 'klienci\\'+klient+'\\2.xls'

    try:
        wb = xlrd.open_workbook(file)
    except Exception:
        logging.info('INFO: Nie mogę otworzyć pliku 2')
        logging.exception("message")
```

```
    try:
        sheet = wb.sheet_by_index(0)
        var = {}
        var['nazwisko'] = sheet.cell_value(1, 3)+' '+sheet.cell_value(1, 2)
        var['plec'] = sheet.cell_value(1, 5)
        logging.info('INFO: Odczyt nazwiska i płci z pliku 2 udany')
    except Exception:
        logging.error('Błąd odczytu nazwiska i płci')
        logging.exception("message")

    try:
        skaner = scanfile2(file)
        logging.info('INFO: Plik 2 zeskanowany')
    except Exception:
        logging.error('Błąd metody scanfile2')
        logging.exception("message")

    try:
        df = pd.read_excel('klienci\\'+klient+'\\2.xls', header=skaner['TABC'][1], nrows =
skaner['TABC'][0])
        s = df.iloc[-1]
        logging.info('INFO: pole TABC przetworzone')
    except Exception:
        logging.error('Błąd pola TABC w pliku 2')
        logging.exception("message")
        pass

    try:
        df2 = pd.read_excel('klienci\\'+klient+'\\2.xls', header=skaner['RISK'][1], nrows =
skaner['RISK'][0], usecols='C:W')
        s2 = df2.iloc[-1]
        s2 = s2.rename(index={'BMI':'BMI_2'})
```

```
        logging.info('INFO: pole RISK przetworzone')
    except Exception:
        logging.error('Błąd pola RISK w pliku 2')
        logging.exception("message")
        pass

    try:
        df3 = pd.read_excel('klienci\\'+klient+'\\2.xls', header=skaner['BMI'][1], nrows = skaner['BMI'][0], 
usecols='C:E')
        s3 = df3.iloc[-1]
        s3 = s3.rename(index={'HEIGHT':'HEIGHT_2','WEIGHT':'WEIGHT_2','BMI':'BMI_3'})
        logging.info('INFO: pole BMI przetworzone')
    except Exception:
        logging.error('Błąd pola BMI w pliku 2')
        logging.exception("message")
        pass

    try:
        df4 = pd.read_excel('klienci\\'+klient+'\\2.xls', header=skaner['BODYFAT'][1], nrows = 
skaner['BODYFAT'][0], usecols='C:D')
        s4 = df4.iloc[-1]
        s4 = s4.rename(index={'FATM':'FATM_2','FATP':'FATP_2'})
        logging.info('INFO: pole BODYFAT przetworzone')
    except Exception:
        logging.error('Błąd pola BODYFAT w pliku 2')
        logging.exception("message")
        pass

    try:
        df5 = pd.read_excel('klienci\\'+klient+'\\2.xls', header=skaner['BODYWATER'][1], nrows = 
skaner['BODYWATER'][0], usecols='C:D')
        s5 = df5.iloc[-1]
```

```
        logging.info('INFO: pole BODYWATER przetworzone')
    except Exception:
        logging.error('Błąd pola BODYWATER w pliku 2')
        logging.exception("message")
        pass

    var['data'] = pd.to_datetime(s['DATETIME'])

    var['birthdate'] = datetime.datetime(*xlrd.xldate_as_tuple(sheet.cell_value(1, 6), wb.datemode))

    try:
        var['wiek'] = np.floor((var['data']-var['birthdate']).days/365.25).astype(int)
    except Exception:
        logging.error('Błąd liczenia wieku')
        logging.error(f"Zmienna data: {var['data']}, typ: {type(var['data'])}")
        logging.error(f"Zmienna birthdate: {var['birthdate']}, typ: {type(var['birthdate'])}")
        logging.exception("message")
        var['wiek'] = None
        pass

    var['dane'] = s[6:]
    var['dane2'] = s2
    var['dane3'] = s3
    var['dane4'] = s4
    var['dane5'] = s5

    return var

def file3(klient):

    try:
```

```
        df = pd.read_excel('klienci\\'+klient+'\\3.xls', header=4, usecols='D:F')
    except Exception:
        logging.error('INFO: Nie mogę otworzyć pliku 3')
        logging.exception("message")

    df.iloc[0,0] = 0
    df = df.dropna(subset=['PRĘDKOŚĆ/MOC'])

    return df



df = pd.DataFrame(dirs, columns=['klient'])
df1 = pd.DataFrame()
df2 = pd.DataFrame()
df3 = pd.DataFrame()

df['LP'] = np.nan
df['Imię i nazwisko (plik nr 1)'] = np.nan
df['Data testu (plik nr 1)'] = np.nan
df['Protokół (plik nr 1)'] = np.nan
df['płeć (plik nr 1)'] = np.nan
df['czas progu AT (plik nr1)'] = np.nan
df['czas progu RC (plik nr 1)'] = np.nan
df['czas max (plik nr 1)'] = np.nan
df['Rodzaj testu'] = np.nan

ii = 1

for i, j in df.iterrows():

    s = f'Klient: {j.klient}'
```

```
print(s)
logging.info(s)


try:
    var = file1(j.klient)
except Exception:
    logging.error('Błąd 1 przetwarzania pliku 1')
    logging.exception("message")
    pass


try:
    df.loc[i,'LP'] = ii
    df.loc[i,'Imię i nazwisko (plik nr 1)'] = var['nazwisko']
    df.loc[i,'Data testu (plik nr 1)'] = var['data_testu']
    df.loc[i,'Protokół (plik nr 1)'] = var['protokol']
    df.loc[i,'płeć (plik nr 1)'] = var['plec']
    df.loc[i,'czas progu AT (plik nr1)'] = var['AT']
    df.loc[i,'czas progu RC (plik nr 1)'] = var['RC']
    df.loc[i,'czas max (plik nr 1)'] = var['Max']
    df.loc[i,'Rodzaj testu'] = var['Rodzaj testu']
    df_a = var['df'].copy()


    if 'Speed' in df_a.columns:
        df_a['PRĘDKOŚĆ/MOC'] = df_a.Speed
    elif 'Power' in df_a.columns:
        df_a['PRĘDKOŚĆ/MOC'] = df_a.Power
    else:
        df_a['PRĘDKOŚĆ/MOC'] = df_a.Load1
    df_a['PRĘDKOŚĆ/MOC'] = df_a['PRĘDKOŚĆ/MOC'].fillna(0).astype(int)
```

```
        delta_pm = df_a['PRĘDKOŚĆ/MOC'].drop_duplicates().diff(1).median()

        df_a['Protokół - delta prędkość/moc'] = delta_pm

        delta_pc = df_a[['PRĘDKOŚĆ/MOC','t']].drop_duplicates(subset=['PRĘDKOŚĆ/MOC'])

        df_a['Protokół - delta czas'] = pd.to_datetime(delta_pc['t'],
format='%H:%M:%S').diff().median().seconds

        df_a['Protokół - prędkość/moc startowa'] = df_a['PRĘDKOŚĆ/MOC'].iloc[0]


        p_AT = df_a[df_a.t==var['AT']]['PRĘDKOŚĆ/MOC'].iloc[0]

        df_AT = df_a[df_a['PRĘDKOŚĆ/MOC']==p_AT]

        t_AT = len(df_AT)

        l_AT = len(df_AT[df_AT.t>var['AT']])

        df_a['Dokładna prędkosc/moc na progu AT'] = p_AT-l_AT*delta_pm/t_AT


        p_RC = df_a[df_a.t==var['RC']]['PRĘDKOŚĆ/MOC'].iloc[0]

        df_RC = df_a[df_a['PRĘDKOŚĆ/MOC']==p_RC]

        t_RC = len(df_RC)

        l_RC = len(df_RC[df_RC.t>var['RC']])

        df_a['Dokładna prędkosc/moc na progu RC'] = p_RC-l_RC*delta_pm/t_RC


        p_Max = df_a[df_a.t==var['Max']]['PRĘDKOŚĆ/MOC'].iloc[0]

        df_Max = df_a[df_a['PRĘDKOŚĆ/MOC']==p_Max]

        t_Max = len(df_Max)

        l_Max = len(df_Max[df_Max.t>var['Max']])

        df_a['Dokładna prędkosc/moc max'] = p_Max-l_Max*delta_pm/t_Max


        df_a['LP'] = ii

    except Exception:

        logging.error('Błąd 2 przetwarzania pliku 1')

        logging.exception("message")

        pass
```

```
    try:
        try:
            var2 = file2(j.klient)
        except Exception:
            logging.error('Błąd metody file2')
            logging.exception("message")

        s_2 = pd.Series()
        s_2['LP'] = ii
        s_2['Imię i Nazwisko (plik nr 2)'] = var2['nazwisko']
        s_2['Płeć (plik nr 2)'] = var2['plec']
        s_2['Data testu (plik nr 2)'] = pd.to_datetime(var2['data'])
        s_2['Wiek (plik nr 2)'] = var2['wiek']
        s_2 = pd.concat([s_2, var2['dane'], var2['dane2'], var2['dane3'], var2['dane4'], var2['dane5']])
        df2 = df2.append(s_2, ignore_index=True)[s_2.index.tolist()]
    except Exception:
        logging.error('Błąd przetwarzania pliku 2')
        logging.exception("message")
        pass

    try:
        df3 = file3(j.klient)
        df3 = df3[df3['PRĘDKOŚĆ/MOC'] == pd.to_numeric(df3['PRĘDKOŚĆ/MOC'], 'coerce') // 1]
        df3['PRĘDKOŚĆ/MOC'] = df3['PRĘDKOŚĆ/MOC'].astype(int)
        df3_pure = df3.copy()

        df1a = pd.DataFrame(index=df_a.index)
        df1a['PRĘDKOŚĆ/MOC'] = df_a.loc[:,'PRĘDKOŚĆ/MOC']

        df3 = pd.merge_asof(df1a, df3[['PRĘDKOŚĆ/MOC','LA (mleczan)']], on='PRĘDKOŚĆ/MOC',
direction='forward')
```

```
    df3.index = df1a.index
    df_a['LA (mleczan)'] = df3['LA (mleczan)']
except Exception:
    df3_pure = None
    logging.error('Błąd przetwarzania pliku 3')
    logging.exception("message")
    pass

try:
    df_AT = progi(df_a, df3_pure, var['AT'], 'Próg AT')
    df_RC = progi(df_a, df3_pure, var['RC'], 'Próg RC')
    df_max = progi(df_a, df3_pure, var['Max'], 'Max')

    df_a = (
            df_a.assign(key=0)
            .merge(df_AT.assign(key=0), how='left', on='key')
            .merge(df_RC.assign(key=0), how='left', on='key')
            .merge(df_max.assign(key=0), how='left', on='key')
            .drop('key', axis=1)
            )

except Exception:
    logging.error('Błąd zliczania mleczanow')
    logging.exception("message")
    pass

try:
    df1 = df1.loc[:,~df1.columns.duplicated()]
    df_a = df_a.loc[:,~df_a.columns.duplicated()]
    df1 = df1.append(df_a, sort=False)
except Exception:
```

```
        logging.error('Błąd 3 przetwarzania pliku 1')
        logging.exception("message")
        pass

    ii+=1

logging.info(f'Rozmiar pliku df: {len(df)}')
logging.info(f'Rozmiar pliku df1: {len(df1)}')
logging.info(f'Rozmiar pliku df2: {len(df2)}')

try:
    df_final = df.merge(df1, how='left', on='LP')
except Exception:
    logging.error('Nie udało się scalić pliku df1')
    logging.exception("message")
    pass

try:
    df_final = df_final.merge(df2, how='left', on='LP')
except Exception:
    logging.error('Nie udało się scalić pliku df2')
    logging.exception("message")
    pass

logging.info(f'Rozmiar pliku df_final: {len(df_final)}')



try:
    df_final['Próg AT - moc generowana relatywnie'] = \
        df_final['Próg AT - Moc generowana (globalnie)']/df_final['WEIGHT']
    df_final['Próg RC - moc generowana relatywnie'] = \
```

```
        df_final['Próg RC - Moc generowana (globalnie)']/df_final['WEIGHT']
    df_final['Max - moc generowana relatywnie'] = \
        df_final['Max - Moc generowana (globalnie)']/df_final['WEIGHT']
except Exception:
    logging.error('Błąd przetwarzania mocy generowanej relatywnie')
    logging.exception("message")
    pass


logging.info('Dane pobrane')
try:
    df_final.to_excel(f'baza_{str(datetime.datetime.now().strftime("%Y%m%d%H%M%S"))}.xlsx')
    logging.info('INFO: Dane wyeksportowane do excela')
except Exception:
    logging.error('Błąd: Nie mogę wyeksportować pliku do excela')
    logging.exception("message")


print('Gotowe')
```
